# Bowel Habits and Functional Constipation in Healthy Children—A Longitudinal Birth‐Cohort Study

**DOI:** 10.1111/apa.70540

**Published:** 2026-04-08

**Authors:** C. Gatzinsky, U. Sillén, M. Bräutigam, C. Kullberg‐Lindh, S. Thornberg, S. Sjöström

**Affiliations:** ^1^ Institute of Clinical Sciences, Department of Paediatrics, Sahlgrenska Academy Gothenburg University Gothenburg Sweden; ^2^ Department of Paediatric Surgery Queen Silvia Children's Hospital Gothenburg Sweden; ^3^ Paediatric Outpatient Clinic Lerum Sweden

**Keywords:** bowel habits, functional constipation, long‐term outcome, Rome 3 criteria

## Abstract

**Aim:**

Functional constipation is common during childhood. Parents' concerns about their child's bowel habits are often the reason for seeking healthcare. We aim to report data on normal bowel habits, prevalence of functional constipation, long‐term outcome, and a search for risk factors in children during the first 2.5 years of life.

**Methods:**

This prospective longitudinal birth‐cohort study included 122 healthy term infants, and repeated questionnaires were answered by parents.

**Result:**

Stool frequency declined with age and stabilised after 6 months. Stool consistencies changed, with hard and very hard stools increasing with age. Functional constipation was found in 22.1%, and at the last follow‐up, 69.6% were still on medication. Relapse was seen in 26.1% on follow‐up. Risk factors at baseline or at 2 months of age that increased the odds of having functional constipation thereafter were not found. Breastfeeding at 2 weeks decreased the probability of FC in the first 2.5 years of life.

**Conclusion:**

We report data on normal bowel habits from birth to 2.5 years of age. One fifth of the children were diagnosed with functional constipation, and relapse was common; therefore, endurance of treatment, repeated visits, and long‐term follow‐up are necessary since many children need medication for several years.

## Summary

- We report data on bowel habits and the prevalence of functional constipation (FC) during the first 2.5 years of life.
- More than one‐fifth of healthy children developed FC, and more than one‐quarter of children with FC experienced a relapse.
- Infants who did not breast‐feed at 2 weeks of age had a higher probability of developing FC during the first 2.5 years of life, compared to infants who breast‐fed.

**Abbreviations**
CI: Confidence interval
DRE: Digital rectal examination
FC: Functional constipation
SD: Standard deviation

## 1 Introduction

Gastrointestinal problems during childhood are a major reason for parents seeking medical attention for their child [1]. One of the most common childhood diagnoses globally is functional constipation (FC) [1, 2, 3], as defined by the Rome IV criteria [4]. Knowledge of normal bowel habits and how they change in healthy children during childhood is important in making a correct diagnosis, but also in avoiding unnecessary investigations, and to reassure parents when changes in their child's bowel habits are simply signs of normal development. Bowel habits in healthy infants and toddlers change during childhood [5, 6, 7, 8, 9, 10], but there is a lack of longitudinal cohort studies and also of data from different countries.

Some evidence indicates that FC is increasing worldwide, with rising healthcare costs as a consequence [11, 12]. Functional Constipation has a great impact on public health [13], and it is associated with a low quality of life [14] and emotional and behavioural problems [15], despite being a benign disorder without an organic cause. Therefore, diagnosing FC is important, and prognosis is dependent on prompt diagnosis and initiation of treatment [16, 17]. Functional constipation can have an early onset, sometimes during infancy [2]. The prevalence of FC during infancy is estimated to be 15%, and in children < 4 years of age, between 1.7% and 29.2% [2, 13]. Peak incidence is believed to occur between 2 and 4 years of age, when potty training starts [18]. The long‐term outcome of childhood FC is not well known. Studies show that about 60% of children can come off medication after one year of treatment [17, 19]. However, up to one‐third of children will continue to have problems beyond puberty [19, 20]. There is a need for longitudinal cohort studies to determine long‐term outcomes, identify predictive factors affecting the outcome and describe prevalence at different ages.

The primary aim of this study was to describe normal bowel habits in a cohort of healthy, full‐term children from Sweden from birth up to 2.5 years of age, and to evaluate the prevalence of FC. We also wanted to evaluate associations between FC and demographic data, search for predictive factors at 2 months of age for developing FC during the first 2.5 years in life, and describe the outcome of FC treatment in this cohort.

## 2 Methods

### 2.1 Study Design

A birth cohort of healthy full‐term infants was prospectively observed from birth to 2.5 years of age as part of a longitudinal study of children followed from infancy to six years of age. The study design and outcomes have been described in detail for the first year of life [21]. At 18 and 30 months, questionnaires were completed at home by the parents, as well as a three‐day bowel diary in which data on bowel habits were reported. Children fulfilling the Rome III criteria for FC [16] were identified. When the study was initiated, the Rome IV criteria [4] were not yet published.

As previously described, the parents contacted the study centre when defecation problems occurred, and organic disease was excluded at a visit to the clinic [21]. If FC was diagnosed, treatment was given to all children according to national guidelines (Figure S1), both with respect to initiation and discontinuation of treatment. All received the same information regarding diet (Figure S1). Treatment success was defined as the absence of Rome III criteria in patients and discontinuation of medication. Children, once diagnosed with FC, remained in the group “children with FC” for the whole study period, regardless of symptoms or medication. Figure S2 clarifies whether or not treatment was ongoing at the time of questionnaires and bowel diary in children with FC.

All children attended the well‐baby clinic according to Swedish guidelines. If a food allergy was suspected, an elimination diet for the breastfeeding mother was recommended. A change in formula feeds or an elimination diet was recommended for the child. Skin prick tests and/or blood samples were not routinely taken. If a child was diagnosed with FC, information was continuously provided by the study centre to the well‐baby clinic.

Given an FC proportion of 15% of the population, a sample size of 120 infants was needed to achieve a 95% confidence interval (CI) for a proportion with a half‐width less than 7.5% with a probability of > 90%.

### 2.2 Subjects

Healthy full‐term newborns from a well‐baby maternity department at a university hospital in Sweden were enrolled in the study between September 2014 and September 2019. Inclusion criteria were the mother's first child, and at least one of the parents was able to understand Swedish. Full‐term was defined as > 36 weeks of gestational age at birth. Exclusion criteria were neonatal illness, surgery, or medication affecting defecation during the study period. The suspicion or presence of a food allergy was not considered an exclusion criterion.

### 2.3 Questionnaires and Three‐Day Bowel Diary

Questionnaires and the three‐day bowel diary during the first year have previously been described in detail [21]. At 18 and 30 months, questions about toilet‐training were added, but the bowel diary remained the same. Toilet‐training was defined as regular attempts to use the toilet or potty, regardless of how often. Being free from diapers was based on parents' conclusions that diapers were no longer necessary. There were also questions regarding the child's preference for a special procedure when defecating to identify withholding behaviour (e.g., standing still with straight stiff legs).

### 2.4 Statistical Analysis

The distribution of continuous variables is given with mean, standard deviation, median, minimum, maximum and 95% CI for the mean. Categorical variables are given as numbers and percentages. For comparison between the two groups of children, with and without FC, Fisher's exact test was used for dichotomous variables. Fisher's non‐parametric permutation test for comparison of means was used for continuous variables. The Mantel–Haenszel chi‐square test was used for ordered categorical variables, and the Pearson chi‐square test for non‐ordered categorical variables. The most important results are the mean differences between the two groups, with 95% confidence interval (CI). From these 95% CIs, it is possible to judge how much better both groups can be compared to the other group, especially with non‐significant tests.

To identify risk factors for having FC at two months, univariable logistic regression was performed. The main results are numbers and percentages of FC in each subgroup and odds ratio (OR) with 95% CI and area under the ROC curve (AUC).

All significance tests were two‐sided and conducted at the 5% level. Statistical Analysis System (SAS) Version 9.4 was used for all analyses.

### 2.5 Ethical Considerations

The study protocol was approved by the Local *Medical Ethical Research Committee* (No. 249–13), and written informed consent was obtained from parents at inclusion.

## 3 Results

### 3.1 Study Population

The study included 122 infants. During the study period, a total of 18 infants dropped out, 15 of which during the first year, and none of them was diagnosed with FC. It was not obligatory to explain reasons for drop‐out; hence, no reason (14/18) was the most common. Moving to another city (2/18), lack of time (1/18), and not wanting to participate in investigations (1/18) were voluntarily reported reasons. See the study flow chart in Figure S3 for details regarding drop‐outs and completed questionnaires. No child was excluded.

### 3.2 Demographics

Baseline characteristics are presented in Table 1. Of all infants, 65/122 (53.3%) were male. The mean gestational age was 40.1 (1.5) weeks, birthweight was 3.5 (0.5) kg, and birth length was 50.4 (2.2 cm). In infants where meconium passage could be dated, the majority 108/114 (94.7%) passed meconium during the first 24 h. Most parents were from Sweden, with the highest educational level being college.

**TABLE 1:** Baseline characteristics in children with and without functional constipation.

Variable	All children (*n* = 122)	Children without functional constipation (*n* = 99)	Children with functional constipation (*n* = 23)	*p*‐value	Difference between groups Mean (95% CI)
Sex					
Male	65 (53.3%)	54 (54.5%)	11 (47.8%)		6.7% (−18.6%; 32.0%)
Female	57 (46.7%)	45 (45.5%)	12 (52.2%)	0.72	−6.7% (−32.0%; 18.6%)
Delivery mode	*n* = 114	*n* = 91	*n* = 23		
Vaginal delivery	88 (77.2%)	66 (72.5%)	22 (95.7%)		
Elective caesarean section	7 (6.1%)	7 (7.7%)	0		
Emergency caesarean section	19 (16.7%)	18 (19.8%)	1 (4.3%)	0.063	
Gestational age [weeks]	40.1 (1.5) 40.3 (36.9; 43) (39.8; 40.4) *n* = 113	40.2 (1.5) 40.1 (36.9; 43) (39.8; 40.5) *n* = 90	40.0 (1.7) 40.6 (37.1; 42.3) (39.3; 40.8) *n* = 23	0.75	0.1 (−0.6; 0.8)
Birth weight [g]	3 518 (521) 3 500 (2 410; 4 830) (3 422; 3 614) *n* = 115	3 519 (517) 3 485 (2 510; 4 830) (3 412; 3 627) *n* = 92	3 511 (547) 3 650 (2 410; 4 543) (3 275; 3 748) *n* = 23	0.95	8.0 (−241.2; 250.4)
Birth length [cm]	50.4 (2.2) 50 (45; 57) (50.0; 50.8) *n* = 111	50.4 (2.2) 50 (45; 57) (49.9; 50.8) *n* = 88	50.6 (2.3) 50 (46; 54) (49.6; 51.5) *n* = 23	0.71	−0.2 (−1.3; 0.8)
Meconium passage within 24 h	*n* = 114	*n* = 93	*n* = 21		
Yes	108 (94.7%)	88 (94.6%)	20 (95.2%)	1.00	−0.6% (−13.7%; 12.5%)
Country of origin of birth parents	*n* = 117	*n* = 94	*n* = 23		
Sweden	98 (83.8%)	78 (83.0%)	20 (87.0%)		
Asia	11 (9.4%)	8 (8.5%)	3 (13.0%)		
Europe	3 (2.6%)	3 (3.2%)	0		
Africa	3 (2.6%)	3 (3.2%)	0		
Nordic countries	1 (0.9%)	1 (1.1%)	0		
South America/Europe	1 (0.9%)	1 (1.1%)	0	0.73	
Highest educational level in the family	*n* = 107	*n* = 85	*n* = 22		
High school	24 (22.4%)	19 (22.4%)	5 (22.7%)		−0.4% (−22.9%; 22.1%)
College	83 (77.6%)	66 (77.6%)	17 (77.3%)	1.00	0.4% (−22.1%; 22.9%)

*Note:* Baseline characteristics of all 122 healthy children included in the study. Divided into children without functional constipation and children with a diagnosis of functional constipation anytime during the first 30 months of life. To be categorised under “Sweden”, one or both parents had to be born in Sweden. Parents had the same country of origin in all families but two, where the combination of South America and Europe was noted. The highest educational level in the family is noted. College is a higher education in the Swedish context. Categorical data are expressed as numbers (percentages) and continuous data as mean (SD), median (max–min), 95% CI for Mean.

Abbreviation: *n* = number of children.

### 3.3 Stool Frequency and Consistency in All Children

**FIGURE 1:**
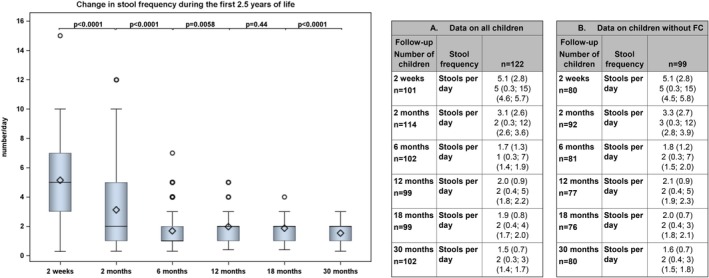
Figure with box plot and table (A) showing stool frequency and how it changes in all children at follow‐ups. Also, table (B) showing data on stool frequencies and how they change in children without functional constipation at any time. *n* (%) is presented for categorical variables. Mean (standard deviation), median (min–max), 95% confidence interval for the mean are shown for continuous variables. *n* = number of children.

In all children, stool frequency declined with age from 5.1 (2.8) stools/day at 2 weeks to 1.9 (0.8) stools per day at 18 months and 1.5 (0.7) stools per day at 30 months (Figure 1). The greatest reduction was seen during the first 6 months. There was a large inter‐individual variation in stool frequency during the first 2 months of life.

**FIGURE 2:**
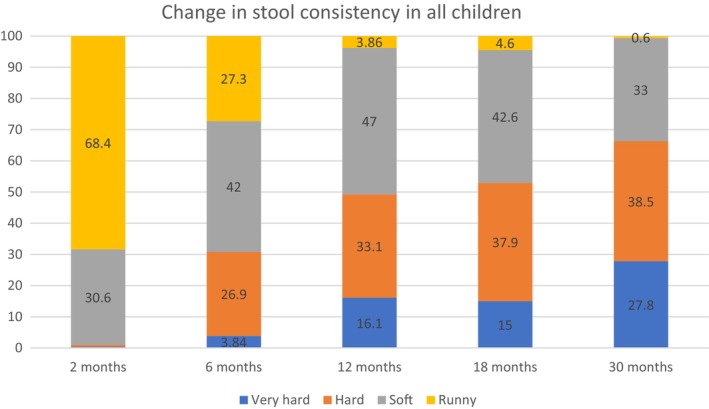
Figure showing the distribution in percentage of stool consistencies at different ages in all children. At 2 months, very hard consistency accounts for 0.4% and hard consistency 0.6%, numbers too small to be visualised in the figure.

Stool consistency changed (Figure 2), with a significant decrease in the percentage of runny stools with increasing age. Hard or very hard stools were uncommon before 6 months of age, but after that, increases in these two categories were seen.

### 3.4 Children With Functional Constipation

During the study period, 23/104 (22.1%) children were diagnosed with FC, with a mean age at diagnosis of 0.94 (0.79) years. Fifteen infants were diagnosed before the age of one, and three of them were less than 2 months old. Another five children were diagnosed between years 1 and 2, and another three children between the ages of 2 and 2.5 years. See Figure S2 for details. There were no differences in demographics between children with or without FC. No demographic data were identified as risk factors for developing FC during the first 30 months of life (Table S1).

Table 2 shows the reported criteria at the time of diagnosis in children with FC. All children reported a history of hard and/or painful bowel movements. In age group 1 (*n* = 15), one third had a palpable faecal mass on digital rectal examination (DRE). In age group 2 (*n* = 8), this was not found, although two children did not have a physical examination at the time of diagnosis due to living in a remote city, and in another two, DRE was not performed due to fear. A history of excessive stool retention was more common in age group 2 (*p* = 0.012).

**TABLE 2:** Reported diagnostic criteria at time of diagnosis in children with functional constipation.

Criteria for functional constipation	All children with functional constipation (*n* = 23)	Age group 1 0–1.0 years (*n* = 15)	Age group 2 1.1–2.5 years (*n* = 8)	*p*‐value	Difference between groups Mean (95% CI)
History of painful or hard bowel movements	23 (100.0%) (85.2%–100.0%)	15 (100.0%) (78.2%–100.0%)	8 (100.0%) (63.1%–100.0%)	N/A	
Presence of a large faecal mass in the rectum*	5 (21.7%) (7.5%–43.7%)	5 (33.3%) (11.8%–61.6%)	0 (0.0%)	0.18	−33.3 (−61.6; 6.9)
Two or fewer defecations per week	18 (78.3%) (56.3%–92.5%)	11 (73.3%) (44.9%–92.2%)	7 (87.5%) (47.3%–99.7%)	0.83	14.2 (−26.5; 46.4)
History of large‐diameter stools that may obstruct the toilet	4 (17.4%) (5.0%–38.8%)	3 (20.0%) (4.3%–48.1%)	1 (12.5%) (0.3%–52.7%)	1.00	−7.5 (−39.5; 32.7)
History of excessive stool retention	8 (34.8%) (16.4%–57.3%)	2 (13.3%) (1.7%–40.5%)	6 (75.0%) (34.9%–96.8%)	0.012	61.7 (14.5; 88.2)
At least 1 episode per week of incontinence after the acquisition of toileting skills	0 (0.0%)	0 (0.0%)	0 (0.0%)	N/A	

*Note:* Table showing children diagnosed with functional constipation (*n* = 23) in the study, divided into age groups, and reported diagnostic criteria at time of diagnosis in each age group. *If children in the older age group fulfilled at least 2 criteria for functional constipation and showed signs of fear, only an anal inspection of the perineum was done at physical examination and not a digital rectal examination. Categorical data are expressed as numbers (percentages) and *n* = number of children. For categorical variables, *n/N* (%) and exact 95% CI is presented.

Parents were offered regular outpatient visits or telephone contacts every 3 to 6 months. The last follow‐up during the study period took place when the children had a mean age of 2.7 years (range from 2.4 to 3.7). The mean follow‐up time from FC diagnosis to study end was 1.8 years (range from 0.9 to 2.7). At the last follow‐up, 16/23 (69.6%) children were still on medication, and 7/23 (30.4%) were off medication and without symptoms. Of all the children diagnosed with FC, 6/23 (26.1%) came off medication, but relapsed and were being medicated at the time of the last follow‐up (Figure S2).

### 3.5 Stool Frequency and Consistency

Figure S2 clarifies whether pharmacological treatment was ongoing at the time of questionnaire and bowel diary. Stool frequency was lower in the group of children diagnosed with FC at 12 months (*n* = 15, *p* = 0.018) and at 30 months (*n* = 23, *p* = 0.028) when compared with children without FC (Table S2). There were no differences in stool consistency between children with or without FC at any time. Frequency or consistency were not risk factors for developing FC during the first 30 months of life (Table S1).

### 3.6 Feeding

**FIGURE 3:**
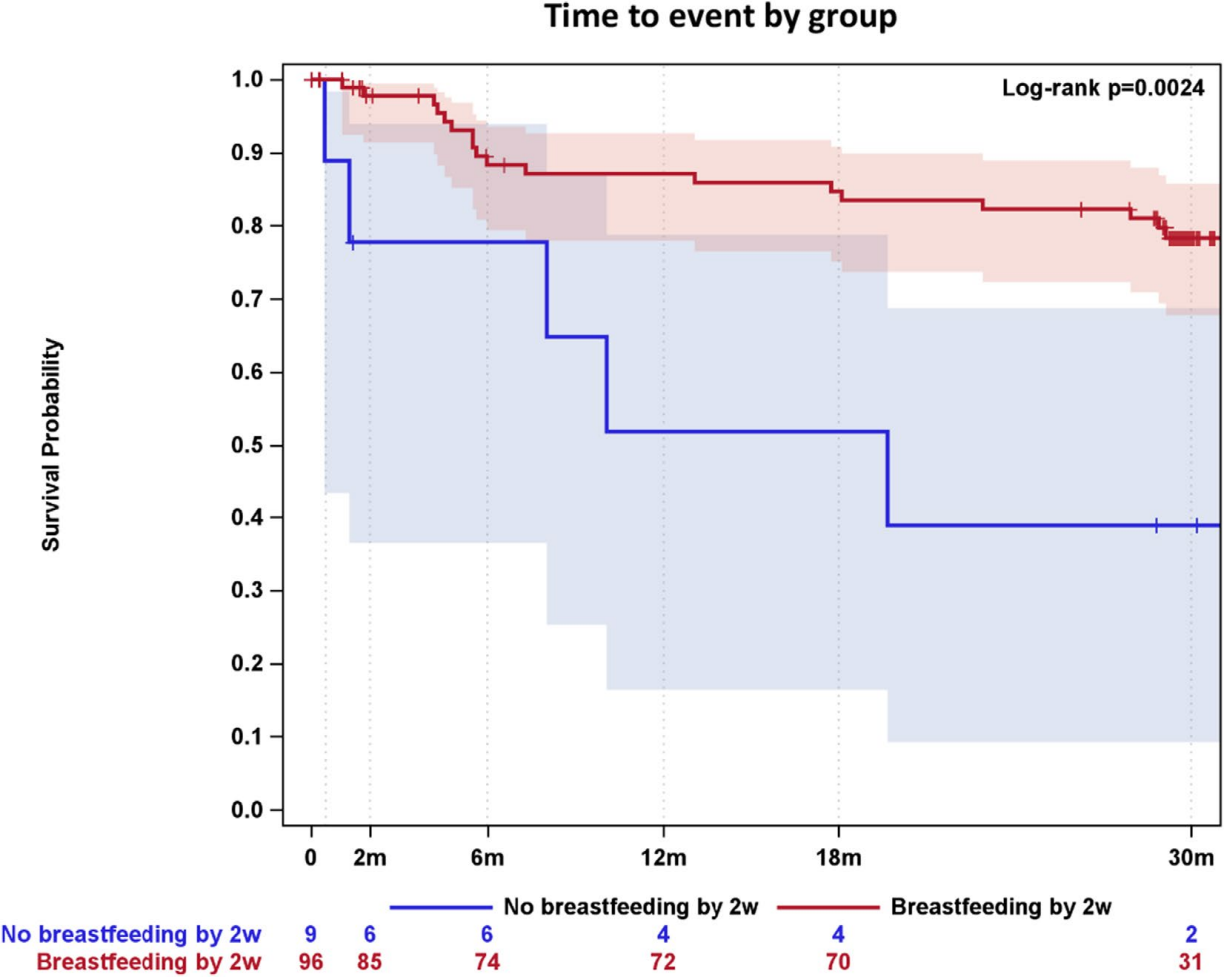
Figure showing a Kaplan–Meier curve of time to event by group. An event means being diagnosed with functional constipation. The group “No breastfeeding by 2 w” includes all children who never had breastmilk between birth and 2 weeks of age (blue). The group “Breastfeeding by 2 w” includes all children that ever had breastmilk between birth and 2 weeks of age (red).

The mean age for first solid foods was 4.4 (0.6) months in all children. After the age of 6 months, all children had solid food. There was no difference in age between children with or without FC at the first solid feeds. The age at the first solid feeds or formula feeding, or breast‐feeding at 2 months were not a risk factor for developing FC during the first 30 months of life (Table S1). In the group of children who had breastmilk between birth and 2 weeks of age, there was a decreased probability of developing FC during the first 2.5 years of life, compared to children who never had breastmilk at any time between birth and 2 weeks (Log‐rank *p* = 0.002) (Figure 3).

### 3.7 Withholding Behaviour

Parents of children with FC more often reported withholding behaviour than in children without FC at 18 months (*n* = 16 (56%) vs. *n* = 89 (18%); *p* = 0.005) and at 30 months (*n* = 23 (59%) vs. *n* = 81 (15%); *p* < 0.001).

### 3.8 Toilet‐Training

The age for starting potty‐training did not differ between children with or without FC (16.4 months vs. 17.3 months; *p* = 0.7), and neither did the age for being toilet‐trained (22.8 months vs. 26.9 months; *p* = 0.071).

### 3.9 Food Allergies

Suspicion of a food allergy was more common in children with FC than in children without FC at 30 months (*n* = 23 (23%) vs. *n* = 81 (4%); *p* = 0.022). All children in whom an allergy was suspected were put on exclusion diets, or their formula was changed, and breastfeeding mothers were recommended to exclude cows' milk from their own diet. No information is available on how many children had a skin prick test or blood samples taken to exclude a milk allergy. Of children with FC, 1/23 (4.3%) had a positive IgE test for cows' milk, reported as additional information by one parent.

## 4 Discussion

We present data from a birth cohort from Sweden in which more than one‐fifth were diagnosed with FC during the first two and a half years of life, and more than half of them had their diagnosis during their first year of life. These findings are in line with a study of Colombian children using the Rome III criteria, with prevalence rates of FC in children < 1 year being 16.1%, and 26.8% at 1 to 4 years [22]. In a study from the US, on the other hand, the prevalence of FC in children aged < 1 year was reported as 4.7%, and in children between 1 and 3 years old, this was 9.4% [23]. The reason for these prevalence differences could be due to different study designs and the use of different questionnaires. Previous studies have also confirmed prevalence differences between geographical regions [2, 24], which could be explained by dietary factors, different parental perceptions and the subjective nature of some of the Rome criteria (e.g., large diameter stools). In our study, one inclusion criterion was that the child was the mother's firstborn, which might result in a higher FC prevalence [22]. In addition, we did not exclude children with suspected or confirmed food allergies. In the group of children with FC, only one was diagnosed by a paediatrician as having a cow's milk allergy. However, diagnosing an allergy to cows' milk in young children is difficult, and more than 50% have a non‐IgE‐mediated allergy, so the true number of children with cows' milk allergy may be higher. Also, we do not have any information for all children as to whether blood samples were taken to exclude cows' milk allergy.

The reported dominating criteria in infants are hard and/or painful bowel movements and rectal impaction [4, 25], in line with our findings. This indicates the importance of performing DRE if only one FC criterion is present. The use of transabdominal ultrasound to detect rectal impaction is appealing, although it does not seem to be useful in infants [26]. We also report that two or fewer stools/week is rather common, in contrast to others [25]. This could be due to a difference in study design. Regarding the toddlers, the dominating criteria were hard and/or painful bowel movements and two or fewer stools/week, consistent with previous findings [4].

Endurance in treatment and follow‐up is crucial in children with FC. At the last follow‐up in this cohort, almost 70% of children with FC at any time were still on medication, and only 30% were without symptoms and laxatives. Data on the long‐term outcome of FC in children is scarce, but several studies suggest that about 60% of children can be cured after 1 year of treatment [17, 19, 27], while 30% of children may continue to have problems into adulthood [19, 20]. The follow‐up time after FC diagnosis in our cohort was a minimum of 0.9 years and a maximum of 2.7 years after FC diagnosis. Van Ginkel et al. showed that in a cohort of children with FC, the number of children without symptoms and laxatives was 45% after 2 years and 50% after 3 years of treatment [19] – numbers which are higher than ours. One reason could be that most of our children had an FC diagnosis before 2 years of age. Successful treatment is reported more often in children in whom FC started after 4 years of age [19], although data are conflicting [17]. Another reason could be the different study populations. The children in Van Ginkel's study were chronically constipated children who had been referred to a tertiary centre, while our cohort were healthy children with FC for a short period.

In our cohort, 26% of the children diagnosed with FC were able to come off initial medication but relapsed and were medicated at the latest follow‐up. Van Ginkel et al. have shown that 50% of children with FC will have a relapse within 5 years [19]. This is, of course, difficult to compare with the results in this study, with its shorter follow‐up time.

We also present longitudinal data on stool frequency and consistency in children without FC, to contribute to knowledge about normal bowel habits in newborns and toddlers [6, 7, 8, 10]. In our cohort, the mean stool frequency at 18 months in children without FC at any time was 2 stools per day. This is comparable with the results from Weaver et al. [5], although they reported data from groups of children at 1 and 2 years of age. Our results lie between, which is consistent with the age‐related decrease in frequency also seen in their study [5]. Fontana et al. [10] reported the mean (SD) stool frequency in a group of children between 1 and 3 years to be 1.4 (0,6) stools per day. This is hard to compare with our results since they did not stratify into age groups, but like us, they found a decrease in frequency with increasing age. To our knowledge, there is only one previous study in which defecation patterns in healthy children > 1 year of age have been studied with a longitudinal design [8]. The mean stool frequencies found in that study by Steer et al. [8] are comparable with ours at 18 months (Steer 1.8 stools/day vs. Gatzinsky 2.0 stools/day) and at 30 months (Steer 1.5 stools/day vs. Gatzinsky 1.6 stools/day). Like us, they also saw a great inter‐individual variability in stool frequencies at young ages.

We found that the number of runny stools decreased with increasing age, in line with previous findings [5]. We also found that hard and very hard stools were uncommon in the first 6 months of life, with an increase at 18 and 30 months, when hard and very hard stools accounted for over 50% of stool consistencies. This is in line with previous findings [8], although neither of the two studies above used the same stool reference chart as us.

As previously reported [21], our data from the first year of life regarding frequency and consistency are consistent with the literature [6, 7, 8, 10].

In children, FC is thought to be the result of an acquired behaviour of withholding of stool after experiencing a painful defecation [4], which has also been confirmed by parents [28]. In our cohort, it was more common in children with FC to have signs of withholding behaviours when defecating, such as standing still with stiff legs, compared to children without FC at 18 and 30 months. Some of the reports from parents included hiding when defecating, a finding not typical of withholding behaviour, although studies have found that this is associated with stool withholding, toilet refusal and FC [29]. Interestingly, withholding behaviour was also reported in children at 12, 18 and 30 months (9.5%, 17.7% and 15.2%) who had not developed FC by the end of the study. Time will tell if these children also develop FC.

We previously reported that breastfeeding at 2 weeks reduced the odds of having FC during the first years of life [21]. We did not find that breastfeeding at 2 months reduced the odds of having FC in the first 30 months of life. However, we could report that in the group of infants who were breast‐fed between birth and 2 weeks of age, there was a decreased probability of developing FC, which supports the thesis that breastmilk is a protective factor early in life [30]. It is known that infant feeding affects the development of gut microbiota and that breast‐fed infants have a different microbiota than formula‐fed infants, whose composition is more adult‐like [31]. When solid foods are introduced and the breast‐fed child is weaned off, the microbiota changes towards a more adult‐like composition. Recent evidence suggests that it is the cessation of breastfeeding that drives this change, rather than the introduction of solids [32], a finding that is in line with our not finding age of first solid feeds a risk factor for developing FC. The role of microbiota and its association with FC have been studied, and proposed mechanisms are that metabolites, cytokines and fermentation may have an osmotic effect or an effect on motility [33, 34]. The microbiota in breast‐fed infants could therefore explain their softer and more frequent stools. On the other hand, the formula‐fed infants with a more adult‐like microbiota have firmer and less frequent stools, which could lead to withholding behaviour and an increased risk of being diagnosed with FC.

Study limitations include the small study population of children with FC and the use of non‐validated questionnaires, although compliant with the Rome III criteria for FC. Also, our study was designed to assess the prevalence of FC and not treatment outcome, so these data might be underpowered. A reason for the lack of identifiable risk factors for developing FC during the first 30 months of life may be due to our small study population. Study strengths include the prospective longitudinal design following a birth cohort, questionnaires conducted as interviews in the first year, which also helped parents understand the questions when completing them at home at 18 and 30 months, and also confirming FC by means of medical history and a physical examination.

In conclusion, this longitudinal study of healthy infants and toddlers shows that FC is a common condition that needs to be addressed and treated properly. More than one‐fifth of healthy children developed FC during the first 30 months of life. Breast milk during the first two weeks of life appears to be a protective factor. More than one quarter of children with FC experienced a relapse during the study period, which underlines that treatment needs to be sustained or repeated, and follow‐up over many years is needed.

## Author Contributions

**C. Gatzinsky:** conceptualization, investigation, writing – original draft, methodology, validation, visualization, writing – review and editing, project administration, formal analysis, software, data curation, supervision, resources. **M. Bräutigam:** investigation, writing – original draft, validation, visualization, writing – review and editing.

## Funding

The study was financed by grants from the Swedish state under the agreement between the Swedish government and county councils, the ALF agreement (ALFGBG‐830501).

## Conflicts of Interest

The authors declare no conflicts of interest.

## Supporting information


**Figure S1:** Figure showing the treatment algorithm for treating functional constipation in the study. The children with functional constipation also had scheduled visits to the outpatient clinic when necessary, besides the study follow‐ups.

**Figure S2:** Figure showing 23 children with functional constipation during the study and their age in years. Coloured bar marks ongoing pharmacological treatment for functional constipation. (1) Lactulose, (2) Polyethylene glycol, (3) Sorbitol‐based rectal micro‐enema (Resulax). The black vertical line indicates when questionnaires and stool diaries are done.

**Figure S3:** Figure showing a study flow chart and the number of infants included at study start and the following check‐ups. At all check‐ups, parents were offered a questionnaire. The number of completed questionnaires at every check‐up is provided. Total drop‐outs during the study period were 18.

**Table S1:** Univariable risk factors at two months of having functional constipation during the first 2.5 years of life.

**Table S2:** Stool frequencies in children with and without functional constipation.

## Data Availability

The data that support the findings of this study are available from the corresponding author upon reasonable request.
