# Supplementary material for: Bowel Habits and Functional Constipation in Healthy Children—A Longitudinal Birth‐Cohort Study
Source: Acta Paediatr. 2026 Apr 8;115(8):1672–80. doi: 10.1111/apa.70540 (PMC13371814; doi:10.1111/apa.70540)
Supplement: Supplementary file 1 — Figure S1: Figure showing the treatment algorithm for treating functional constipation in the study. The children with functional constipation also had scheduled visits to the outpatient clinic when necessary, besides the study follow‐ups. [file APA-115-1672-s003.docx]

| Treatment algorithm for Functional Constipation in children | | |
| --- | --- | --- |
| **Step 1** | Non-pharmacological treatment  This step is repeated at every visit | 1. Parent education 2. Information on potty-training 3. Information on dietary habits: intake of water and fiber should be the age-recommended amount, not more or less 4. Recommendation to give e.g. fruit purees: prune purée, kiwi fruit and grapes when suitable for age |
| **Step 2** | Initial pharmacological treatment | 1. Faecal disimpaction using sorbitol-based rectal micro-enema (Resulax®) if needed 2. Oral Lactulose if < 6 months of age, individualised dose 3. Oral Polyethylene glycol if > 6 months of age or if Lactulose does not work, individualised dose |
| **Step 3** | Maintenance pharmacological treatment | 1. Maintenance treatment with individualised dose for at least 2 months and/or until no ROME III criteria are present 2. Continue treatment when no symptoms are present for at least 1 month |
| **Step 4** | Discontinuation of treatment | 1. Reduce dosage over a period of 1-2 months 2. Contact with MD during this period; if failure, go back to step 3 3. If success continue follow-up in outpatient clinic |

**Figure S1**
