# Supplementary material for: Bowel Habits and Functional Constipation in Healthy Children—A Longitudinal Birth‐Cohort Study
Source: Acta Paediatr. 2026 Apr 8;115(8):1672–80. doi: 10.1111/apa.70540 (PMC13371814; doi:10.1111/apa.70540)
Supplement: Supplementary file 4 — Table S1: Univariable risk factors at two months of having functional constipation during the first 2.5 years of life. [file APA-115-1672-s005.docx]

**Table S1. Univariable risk factors at two months of having functional constipation during the first 2.5 years of life**

| **Variable at 2 month follow-up** | **n** | **Value** | **n (%) of event** | **OR (95%CI) FC diagnosis** | **p-value** | **Area under ROC-Curve (95%CI)** |
| --- | --- | --- | --- | --- | --- | --- |
| **Sex** | 119 | **male** | 9 (14.3%) |  |  |  |
|  |  | **female** | 11 (19.6%) | 1.47 (0.56-3.85) | 0.44 | 0.55 (0.43-0.67) |
| **Delivery mode** | 111 | **vaginal** | 19 (22.4%) | 1.00 | 0.25*** |  |
|  |  | **elective c-sec** | 0 (0.0%) | 0.23 (0.01-5.06)F | 0.35F |  |
|  |  | **emergency c-sec** | 1 (5.3%) | 0.28 (0.05-1.64)F | 0.16F | 0.61 (0.55-0.68) |
| **Gestational age [weeks]** | 110 | **36.86-<40.00** | 9 (20.5%) |  |  |  |
|  |  | **40.00-<41.00** | 4 (16.7%) |  |  |  |
|  |  | **41.00-43.00** | 7 (16.7%) | 0.89 (0.65-1.22) | 0.46 | 0.54 (0.40-0.69) |
| **Birth weight [g]** | 112 | **2410-<3500** | 9 (16.1%) |  |  |  |
|  |  | **3500-<4200** | 10 (23.3%) |  |  |  |
|  |  | **4200-4830** | 1 (7.7%) | 0.98 (0.89-1.07) | 0.64 | 0.51 (0.36-0.66) |
| **Age at first solid feeding [months]** | 101 | **3-<4** | 0 (0.0%) |  |  |  |
|  |  | **4-<5** | 12 (17.4%) |  |  |  |
|  |  | **5-6** | 7 (22.6%) | 1.04 (0.46-2.35) | 0.92 | 0.53 (0.41-0.64) |
| **Highest educational level in the family** | 104 | **high school vs high school** | 4 (17.4%) | 1.00 | 0.90*** |  |
|  |  | **college vs high school** | 15 (18.5%) | 1.08 (0.32-3.64) | 0.90 | 0.51 (0.40-0.61) |
| **Formula feeding (@2m)** | 114 | **no** | 12 (20.7%) |  |  |  |
|  |  | **yes** | 8 (14.3%) | 0.64 (0.24-1.71) | 0.37 | 0.56 (0.43-0.68) |
| **Breast-feeding (@2m)** | 114 | **no** | 6 (27.3%) |  |  |  |
|  |  | **yes** | 14 (15.2%) | 0.48 (0.16-1.43) | 0.19 | 0.56 (0.46-0.67) |
| **Special food for softening stools** | 97 | **no** | 16 (18.2%) |  |  |  |
|  |  | **yes** | 1 (11.1%) | 0.56 (0.07-4.82) | 0.60 | 0.52 (0.45-0.59) |
| **Parents helping the child to defecate** | 113 | **no** | 16 (17.2%) |  |  |  |
|  |  | **yes** | 4 (20.0%) | 1.20 (0.35-4.08) | 0.77 | 0.51 (0.42-0.61) |
| **Stool frequency (numbers/day)** | 111 | **0.3-<1** | 4 (21.1%) |  |  |  |
|  |  | **1-<4** | 10 (20.4%) |  |  |  |
|  |  | **4-12** | 5 (11.6%) | 0.88 (0.70-1.09) | 0.24 | 0.60 (0.46-0.74) |
| **Stool consistency 1 [%] (Very hard)** | 94 | **0.0-<5.3** | 17 (18.7%) |  |  |  |
|  |  | **5.3-25.0** | 1 (33.3%) | 0.81 (0.09-7.48) | 0.86 | 0.49 (0.43-0.54) |
| **Stool consistency 2 [%] (Hard)** | 94 | **0.0-<11.8** | 16 (17.6%) |  |  |  |
|  |  | **11.8-25.0** | 2 (66.7%) | 2.34 (0.76-7.22) | 0.14 | 0.55 (0.47-0.62) |

Table showing univariable risk factors at two months of age for having functional constipation at any time during the first 30 months in life. n= number of infants with an answer to the measured variable. n*= number of infants with functional constipation at any time after 2 months of age during the first 30 months of life for every variable. P-values, OR and Area under ROC-curve are based on original values and not on stratified groups. OR is the ratio for the odds of an increase of the predictor of one unit. ***= p-value for the entire effect/factor/variable.
