# Supplementary material for: Bowel Habits and Functional Constipation in Healthy Children—A Longitudinal Birth‐Cohort Study
Source: Acta Paediatr. 2026 Apr 8;115(8):1672–80. doi: 10.1111/apa.70540 (PMC13371814; doi:10.1111/apa.70540)
Supplement: Supplementary file 5 — Table S2: Stool frequencies in children with and without functional constipation. [file APA-115-1672-s004.docx]

**Table S2. Stool frequencies in children with and without functional constipation**

| **Variable** | **All children** | **Children without functional constipation** | **Children with functional constipation** | **p-value** | **Difference between groups Mean (95% CI)** |
| --- | --- | --- | --- | --- | --- |
| **12 months** | **n=105** | **n=90** | **n=15** |  |  |
| Stools per day | 1.97 (0.91) 2 (0.4; 5) (1.79; 2.15) n=99 | 2.06 (0.92) 2 (0.4; 5) (1.86; 2.26) n=84 | 1.48 (0.70) 1 (0.6; 3) (1.09; 1.87) n=15 | 0.018 | 0.581 (0.100; 1.100) |
| **18 months** | **n=105** | **n=89** | **n=16** |  |  |
| Stools per day | 1.87 (0.78) 2 (0.4; 4) (1.71; 2.02) n=99 | 1.93 (0.73) 2 (0.4; 4) (1.77; 2.09) n=83 | 1.56 (0.93) 1.5 (0.4; 3) (1.07; 2.06) n=16 | 0.084 | 0.366 (-0.047; 0.786) |
| **30 months** | **n=104** | **n=81** | **n=23** |  |  |
| Stools per day | 1.53 (0.72) 2 (0.3; 3) (1.39; 1.67) n=102 | 1.61 (0.68) 2 (0.4; 3) (1.46; 1.76) n=80 | 1.25 (0.80) 1 (0.3; 3) (0.89; 1.60) n=22 | 0.028 | 0.366 (0.037; 0.693) |

Table showing data on stool frequencies in all children and in children with or without functional constipation at any time during the study at different ages. Categorical data are expressed as numbers (percentages) and continuous data as mean (SD), median (max-min), 95% CI for Mean, n=number of children.
